# Experience-based suggestions for improving accessibility of minibus taxis for wheelchair users

**DOI:** 10.4102/ajod.v14i0.1699

**Published:** 2025-08-30

**Authors:** Jerome P. Fredericks, Surona Visagie, Lana van Niekerk

**Affiliations:** 1 Department of Occupational Therapy, Faculty of Medicine and Health Sciences, Stellenbosch University, Cape Town, South Africa; 2 Centre for Disability and Rehabilitation Studies, Faculty of Medicine and Health Sciences, Stellenbosch University, Cape Town, South Africa; 3 Division Occupational Therapy, Department of Health and Rehabilitation; Faculty of Medicine and Health Sciences, Stellenbosch University, Cape Town, South Africa

**Keywords:** wheelchair users, minibus taxi drivers, minibus taxis, caregivers, access, accessibility, transport, co-operative inquiry, peri-urban, marginalised

## Abstract

**Background:**

Accessible transport is a prerequisite for the sustainable developmental goals (SDGs). Without transport, SDGs dependent on community mobility such as health and well-being, education, work and economic growth cannot be achieved.

**Objectives:**

Presenting experience-based suggestions offered by stakeholders to facilitate accessible minibus taxi services for wheelchair users in the peri-urban setting.

**Method:**

The study setting was Paarl-East, Western Cape province of South Africa. A cooperative inquiry methodology was used. Adult wheelchair users (*n* = 9) and their caregivers (*n* = 8), minibus taxi drivers (*n* = 7) and stakeholders (*n* = 4) involved in disability matters in the setting participated. Data were collected in 16 group sessions. Inductive thematic analysis was performed.

**Results:**

Five themes emerged. Theme 1: The ideal communication platform; the need and purpose of databases of wheelchair users and minibus taxi drivers; the use of social media as a communication platform. Theme 2: Fair economical fares focus on service affordability and payment options. Theme 3: Facilitating ideal behaviour patterns discusses the development of mutual respect. Theme 4: Customised minibus taxis highlight the need for a fleet of minibus taxis with different specifications to address different wheelchair users’ needs. Theme 5: Minibus taxi service delivery considerations describes practical strategies such as home pickups and drop-offs.

**Conclusion:**

Current suggestions for solutions need further refinement. Accountability and funding are underexplored.

**Contribution:**

Presenting experience-based suggestions by stakeholders on facilitating accessible minibus taxi services for wheelchair users.

## Introduction

Accessible transport is an important prerequisite for achieving the Sustainable Developmental Goals (SDGs) and key in fulfilling SDG 11.2 ‘affordable, and sustainable transport systems’ for all. Without transport, other SDGs that are dependent on community mobility, such as health and well-being, education, work and economic growth cannot be achieved. The importance of accessible transport was first emphasised by the United Nations Convention on the Rights of Persons with Disabilities (UNCRPD) in 2006 (Nizar 2011). Accessible transport for all is also crucial to the South African National Developmental Plan as it can help to support economic growth, reduce inequality and improve the quality of life of all South Africans (Mohamad Taghvaee et al. 2023). Access to transport is one of the dimensions of pillar 1 of the White paper on the Rights of Persons with Disabilities (Department of Social Development [DSD] 2016).

Despite awareness of the need for inclusion of persons with disabilities as well as policy and legislation advocating for inclusive transport going back 20 years the transportation needs of South African wheelchair users are still neglected (Duri & Luke 2022; Fredericks, Visagie & Van Niekerk 2024a; Lister & Dhunpath 2016). Wheelchair users are often unable to access their communities or various services because of transport and mobility challenges (Duri & Luke 2022; Lister & Dhunpath 2016; Visagie, Visagie & Fredericks 2023). In seeking solutions for these challenges, the voices of wheelchair users have often gone unheard because of marginalisation that result from being a minority group affected by dual vulnerabilities of disability and poverty (Makomborero 2022).

Minibus taxis are the most readily used mode of public transport in South Africa’s sprawling peri-urban settings (Venter 2011). Previous research has shown that wheelchair users face multiple barriers when accessing minibus taxis. Barriers include getting to pick up points, getting into and out of the vehicle, affordability, safety and negative attitudes of drivers and co-commuters alike (Cawood & Visagie 2015; Duri & Luke 2022; Fredericks et al. 2024a; Gudwana 2019; Kett, Cole & Turner 2020; Lister & Dhunpath 2016; Vergunst et al. 2015; Visagie et al. 2023). Research on possible solutions is limited. Kett et al. (2020) summarised various strategies that could or have been used in middle- and low-income countries (LMICs) to enhance the accessibility of transport for persons with disabilities. Transportation applications support flexible schedules and door-to-door services, which are more expensive compared to regular mass rapid transport (MRT) services. In some South African cities, people with disabilities can use a smart card to access state provided public transport systems (Lister & Dhunpath 2016). However, difficulty in locating the machines and impatient drivers has hampered its use (Lister & Dhunpath 2016).

Both MRT and Special Transport System (STS) are in use in the City of Cape Town, a metropolitan area, less than 100 kms from the current study setting. However, Dial-a-Ride, which is a STS, that provides door-to-door transport service to persons with disabilities (Paquette et al. 2007), experiences multifaceted challenges related to registration, unresponsive call centres, scheduling, high demand, a lack of punctuality and little flexibility (Morta-Andrews 2018). In addition, STS are more costly and segregate compared to those that include persons with disabilities (Grisé et al. 2019).

Regarding MRT, the MyCiTi bus service, which was introduced in 2010 in Cape Town, is the only public transport system in Cape Town that adheres to the requirements of universal design and accommodates the needs of wheelchair users. The current study setting of Paarl is not included in the geographical areas where this service has been rolled out (City of Cape Town 2013), or will be in the foreseeable future. As such, minibus taxis remain the most feasible public transport option for persons with disability in the Paarl region.

## Research design and methodology

### Study design

**FIGURE 1:**
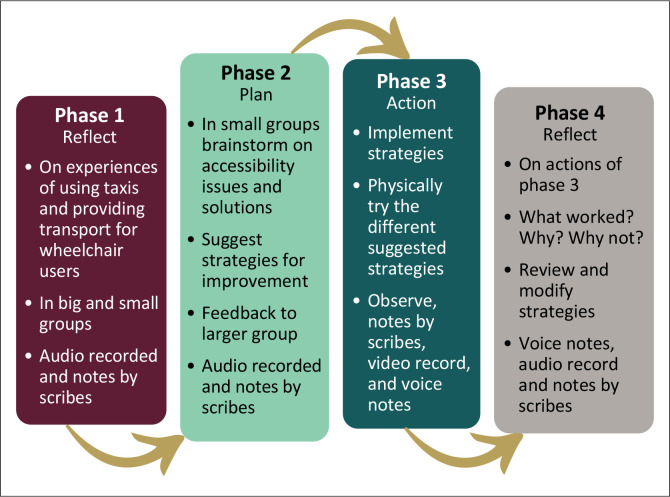
An illustration of the co-operative inquiry phases.

Two groups which are often antagonistic towards each other, namely, wheelchair users and minibus taxi drivers, came together in this cooperative inquiry to identity resolutions advantageous to both groups. Co-operative inquiry was chosen because it draws on the experience and knowledge of co-researchers to make meaning and develop interventions. Co-operative inquiry is cyclic in nature with four phases in each research cycle (Heron 2014; Wooltorton et al. 2020). Figure 1 illustrates the phases for each of the four cycles of this study.

**FIGURE 2:**
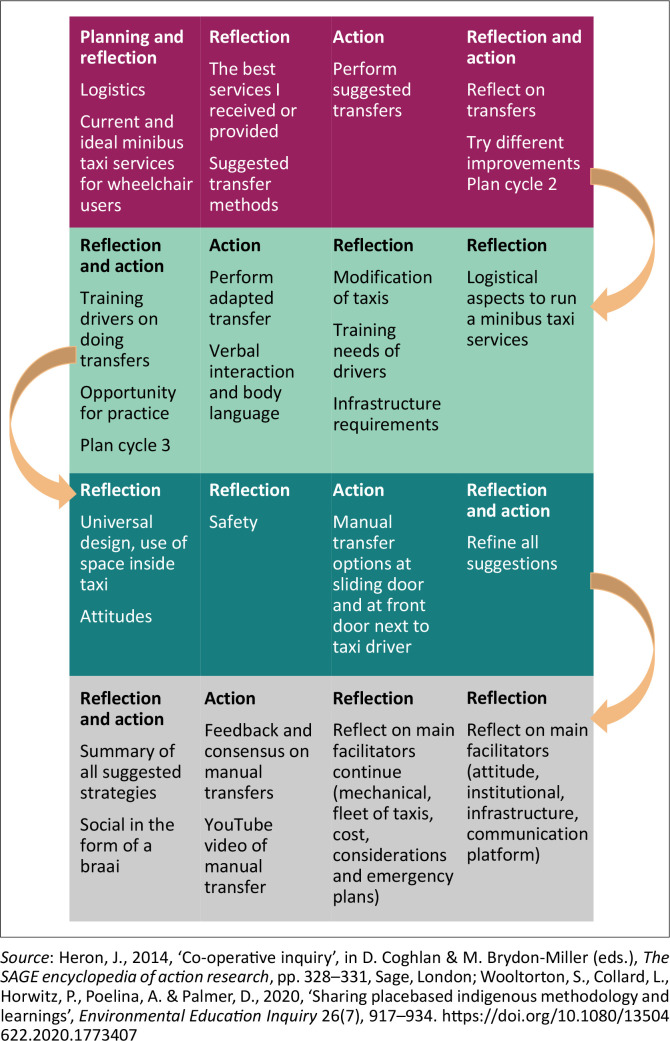
A summary of the processes in the 16 sessions.

Co-operative inquiry cycles built on each other to refine and improve the trustworthiness of the established knowledge (Heron 2014; Wooltorton et al. 2020). Authentic knowledge can only be developed if the co-operative inquiry co-researchers and research team members are jointly responsible for the process and outcomes achieved. The voices of all co-researchers must carry equal weight (Heron 2014). Achieving this level of engagement among co-researchers depends on practical arrangements that are clearly communicated and acceptable, co-researchers’ understanding the steps to be followed and purpose of a specific co-operative inquiry, dealing with hostilities, bonding and developing mutual trust to form a cohesive group (Fredericks et al. 2024b). The actual topics reflected and acted on during this co-operative inquiry are presented in Figure 2.

### Setting

Paarl-East in the Western Cape province of South Africa is a peri-urban area. The majority of Paarl-East’s residents experience severe poverty. Low educational levels and economic struggles have plagued the community. Crime, teenage pregnancy and not completing school are common. Drug-related crimes that negatively impact human development, through its corrosive effects on family structures, health, community economy and safety, reduces the quality of life in the area (Drakenstein Municipality 2017).

The main modes of community mobility in Paarl-East are minibus taxis and/or walking. The town is surrounded by mountains and steep inclines are common. Winters in Paarl are cold and wet, summers hot and dry. Gravel roads and potholes in tarred roads are common in the setting.

Three minibus taxi unions operate in the Paarl area. Together they have formed the Paarl Taxi Forum. Minibus taxi drivers from unions outside Paarl also want to provide their services in the Paarl area, which has led to conflict and violence.

### Population, sampling and recruitment

Co-operative inquiry involves a significant time and personal commitment from co-researchers. Thus, persons who were prepared to commit were sought. The co-researchers who participated in the study came from five different groups:

- Wheelchair users using or wanting to use minibus taxi services.
- Care givers of wheelchair users.
- Minibus taxi drivers.
- Stakeholders involved in disability matters in the setting.
- The first author and the research assistant.

The 30 co-researchers were identified and recruited as follows:

- *Nine wheelchair users* were recruited. Three of them were known to the first author. Four were identified through disabled people’s organisations, and two were sampled following a street-based approach.
- *Eight carers* of these nine wheelchair users.
- *Seven taxi drivers* were approached and recruited through the Paarl Taxi Associated Group.
- Four additional *stakeholders*, consisted of a professional nurse who provided orthopedic services to the community, a disability activist, an interested community member and a community member with technical knowledge pertaining to wheelchairs.
- *The principal investigator* (first author of this article) and the research assistant.

### Data collection

Data were collected between June and December 2021 through 16 co-operative inquiry sessions in four cycles. Each session lasted between 60 and 120 min. The planning and reflection sessions were digitally audio-recorded and four co-researchers (S2, C4, TD4 and C5; see Table 2) kept handwritten notes. The practical sessions were recorded by a professional videographer. After each session, a summary was prepared by the first author and shared with co-researchers. The WhatsApp platform was used for communication. With the permission of the group, all WhatsApp messages were included as data. The reflective journal of the first author also served as a further source of data (Fredericks et al. 2024a).

### Data management and analysis

Data were transcribed and provisionally analysed as the research unfolded to inform subsequent phases and cycles. Video recordings assisted to identify participants, added depth, for example, facial expressions, and body language, as well as provide visual descriptions of physical activities such as boarding a taxi. In developing the themes, Braun and Clarke’s (2012) six step inductive thematic analysis approach was used. Manual line-by-line coding was done by the first author. Codes were organised into meaningful groups that represented provisional themes. The second author was provided with this provisional analysis of the data. Together the authors further developed the themes through an iterative reviewing and refining process (Fredericks et al. 2024a).

### Trustworthiness of the study

Repetitious cycles, of reflection and action following each other, refined understanding and results. Saturation was achieved after the third cycle. A fourth cycle was completed to further the credibility of the findings. Triangulation of methods and data sources further supported credibility, confirmability and dependability. Moreover, iterative analysis and consensus by two authors (JF & SV) further support credibility. To support transferability, the study context and methods were clearly described (Fredericks et al. 2024a; Nowell 2017). The first author kept a reflective diary to record the research process and capture feelings, thoughts, concern and questions to bracket his opinions and allow the findings to reflect the opinions of co-researchers.

### Ethical considerations

The Health Research Ethics Committee of Stellenbosch University (S21/01/009) provided ethical approval on 22 April 2021. Written informed consent was provided by all co-researchers. A first aid officer was on standby during the action phases in case any injuries occurred. His services were not needed. Cash payments and hot meals were used to compensate co-researchers. Transport to attend the sessions was provided. The transcriber signed a declaration safeguarding personal details of participants. For ethical reasons, no video recordings were made of the planning and reflection sessions. The video recordings were viewed by the first author only and some of the video material was shared with the supervisors. Data are stored in the password-protected Stellenbosch University’s SUNScholar Research Repository (where it will be kept for five years).

## Findings

### Demographic details of co-researchers

Seven of the nine wheelchair users were men and their age ranged from 32 to 67 years (Table 1). All of them used orthopaedic style folding frame wheelchairs with no modifiable features. While five of them could propel the wheelchair by themselves, outdoors they all needed assistance to access minibus taxis. Six of the caregivers were women. They were aged between 28 and 67 years. All the taxi drivers were men and their age ranged between 32 and 48 years (Table 2) (Fredericks et al. 2024a).

**TABLE 1:** Demographic details of wheelchair users (co-researchers).

Co-researchers	Gender	Age	Diagnosis	Self-propel or attended propelled	Dependent on assistance to use minibus taxi	Occupation	Income: Source or amount per month	Marital status
WCU1	Male	50	Spinal cord injury	Self-propelled	Dependent	Unemployed	Social grant R2080 US$117.64	Married
WCU2	Female	49	Cerebral vascular accident	Self-propelled with hand and foot	Dependent	Unemployed	Social grant R2080 US$117.64	Married
WCU3	Male	52	Cerebral vascular accident	Self-propelled with hand and foot	Dependent	Unemployed	Social grant R2080 US$117.64	Married
WCU4	Male	57	Cerebral vascular accident	Attended propelled	Dependent	Unemployed	Social grant R2080 US$117.64	Single
WCU5	Male	55	Spinal cord injury	Self-propelled	Dependent	Unemployed	Social grant R2080 US$117.64	Married
WCU6	Male	32	Spinal cord injury	Attended propelled	Dependent	Unemployed	Social grant R2080 US$117.64	Married
WCU7	Female	54	Amputation of the lower limb	Self-propelled	Dependent	Unemployed	Social grant R2080 US$117.64	Single
WCU8	Male	67	Spinal cord injury	Attended propelled	Dependent	Unemployed	Social grant R2080 US$117.64	Married
WCU9	Male	37	Spinal cord injury	Attended propelled	Dependent	Employed	SAP R15 533.97 US$803.91	Married

Source: Fredericks, J.P., Visagie, S. & Van Niekerk, L., 2024a, ‘A qualitative exploration of community mobility experiences of wheelchair users’, *African Journal of Disability* 13, 1253. https://doi.org/10.4102/ajod.v13i0.1253

WCU, Wheelchair user, SAP, South African Police.

**TABLE 2:** Demographic details of other co-researchers.

Co-researchers	Gender	Age (years)	Profession	Income source	Amount per month	Marital status
**Caregivers**						
C1	Female	63	Retired	Social grant	R2080 US$109.23	Divorced
C2	Male	51	Employed	Tradesman	R10 90 055 US$578.82	Married
C3	Female	45	Unemployed	No income	-	Single
C4	Male	28	Unemployed	No income	-	Single
C5	Female	48	Unemployed	No income	-	Married
C6	Female	67	Retired	Social grant	R2080 US$109.23	Married
C7	Female	55	Unemployed	No income		Married
C8	Female	41	Employed	Casual worker	Not disclosed	Married
**Minibus taxi drivers**						
TD1	Male	44	Employed	Taxi driver	R846 703 US$449.6	Married
TD2	Male	32	Employed	Taxi driver	R846 703 US$449.6	Single
TD3	Male	48	Employed	Taxi driver	R846 703 US$449.6	Married
TD4	Male	45	Employed	Taxi driver	R846 703 US$449.6	Married
TD5	Male	48	Employed	Taxi owner/driver	Not disclosed	Married
TD6	Male	38	Employed	Taxi driver	R846 703 US$449.6	Single
TD7	Male	37	Employed	Taxi driver	R846 703 US$449.6	Single
**Other**						
S1	Male	66	Retired	Social grant/activist for disability matters	R2080 US$109.23	Single
S2	Female	62	Retired	Social grant/retired professional nurse	R2080 US$109.23	Divorced
S3	Male	43	Employed	Local government	Not disclosed	Married
S4	Male	34	Employed	Local government	Not disclosed	Married
Primary Investigator (PI)	Male	45	Employed	Lecturer	Not disclosed	Married
Research Assistant (RA)	Male	50	Employed	Lecturer	Not disclosed	Married

*Source*: Fredericks, J.P., Visagie, S. & Van Niekerk, L., 2024a, ‘A qualitative exploration of community mobility experiences of wheelchair users’, *African Journal of Disability* 13, 1253. https://doi.org/10.4102/ajod.v13i0.1253

C, caregiver; TD, taxi driver; S, stakeholder.

### Emerging themes

Five themes, shown in Table 3, emerged from the analysis. Theme 1: ‘The ideal communication platform’ explains the need and purpose of a database of wheelchair users and minibus taxi drivers. It also explores how social media can be used as a communication platform between wheelchair users and minibus taxi drivers. Theme 2: ‘Fair economical fares’ focuses on the ideal price wheelchair users should be paying for using minibus taxi services. Theme 3: ‘Facilitating ideal behaviour patterns’ centres on what can be done to develop a more positive attitude among minibus taxi drivers, wheelchair users and fellow commuters. Theme 4: ‘Customised minibus taxis’ presents the need for a fleet of minibus taxis with different specifications that can address different needs of wheelchair users. Theme 5: ‘Minibus taxis service delivery considerations’ focuses on practical strategies to ponder when providing minibus taxi services to wheelchair users, such as home pickups and drop-offs.

**TABLE 3:** Themes and categories that emerged from the data.

Theme 1: The ideal communication platform	Theme 2: Fair economical fares	Theme 3: Facilitating ideal behaviour patterns	Theme 4: Customised minibus taxis	Theme 5: Minibus taxi service delivery considerations
Database	Free of charge services	Attitudes of drivers, users, and fellow commuters	Fleet with different specs	Handling of wheelchair users during transfers
Communication platform	Same charges Subsidy/contract Drivers decide if and what wheelchair users pay	Education Drivers’ role in education	Modifications of minibus taxis Storing wheelchair	Personal hygiene Inclusiveness Home pickups and drop-offs

#### Theme 1: The ideal communication platform

Co-researchers revealed the need for a database and a communication platform. They envisaged that improved communication would assist taxi drivers to better serve the market offered by wheelchair users.

The **database** was foreseen to serve two purposes. It could provide information on the location, level of assistance required and accommodation needs of wheelchair users who will be using minibus taxi services. It could also offer information on minibus taxi drivers who are willing to transport wheelchair users, including contact and vehicle accessibility related information:

‘… identify the wheelchair users who use or want to use of minibus taxi services, as well as the minibus taxi operators and drivers that are willing to provide minibus taxi services for wheelchair users so that there can be a database of wheelchair users and minibus taxi drivers of the area.’ (S4, Male, 34, Employed)

‘I agree with the database because it will provide us minibus taxi drivers information on where the wheelchair users are located, what their transfer needs are and what assistance can be expected from us. The database for minibus taxi drivers should include their minibus taxi company or minibus taxi owner, the driver’s name and surname, cell phone contact details, and if the driver received training to transport wheelchair users.’ (TD2, Male, 32, Employed)

With regards to databases, it was not clear who should be responsible for the developing and maintaining these databases. Suggestions included a local organisation for persons with disability as well as health authorities at provincial and municipal levels.

With regards to communication platforms, the co-researchers expressed the need for this communication platform on which to book trips and share pick up times. This could reduce long waiting times that were associated with exposure to harsh weather conditions, safety risks and the possibility of bladder and bowel accidents:

‘There is a need to develop a mobile application or forming a WhatsApp group to allow wheelchair users and minibus taxi drivers to communicate with one and another.’ (WCU6, Male, 32, Spinal cord injury)

A dedicated application might have been useful, but concerns were raised about the challenges that come with designing and downloading applications:

‘The mobile app will have to be developed, which comes with its own challenges … in some instances people struggle to download applications.’ (TD5, Male, 48, Employed)

The group decided that a social media application platform can serve as communication platform because people are usually familiar with it. It provides a user friendly and cheap way to communicate:

‘The WhatsApp platform will work best. It is practical and user friendly. Most people are already using it.’ (WCU3, Male, 52, Cerebral vascular accident)

‘I am also in agreement with the WhatsApp application group but regards confidentiality, wheelchair users don’t need to communicate on the group but can also communicate directly with the minibus taxi driver. Because if we all are one group it is going to take too much data, and I don’t want everyone to see my coming and goings.’ (WCU7, Female, 54, Amputation of the lower limb)

Theme 1 focused on the need for communication strategies between wheelchair users and minibus taxi drivers. The discussion ranged from the need for databases to a mobile communication platform. Co-researchers felt databases might help wheelchair users to identify taxi drivers willing to support them and equip taxi drivers with knowledge about the specific needs of the wheelchair users they will provide transport to.

#### Theme 2: Fair economical fares

The group discussed the challenges of an affordable service for wheelchair users versus the taxi driver’s need to make a decent wage:

‘The reality of the taxi driver’s economic needs remain […] the minibus taxi drivers also have families they must take care of.’ (WCU7, Female, 54, Amputation of the lower limb)

Co-researchers debated options including free services, no concession to wheelchair users, allowing the taxi driver to determine the price and subsidised services.

**Free of charge services:** Some co-researchers argued that wheelchair users who receive a social grant should not pay for minibus taxi services. They equated this with not paying for medical services received at government institutions:

‘I would say that pensioners and persons who receives [*a social grant*] or a disability grant should not pay for minibus taxi services. This is based on the principle of the Department of Health that provide free services for pensioners and disabled people who received disability grants.’ (S1, Male, 66, Retired)

**Same charges:** Some group members, especially the taxi drivers, reasoned that if wheelchair users wanted equal rights they should be treated as equals and therefore pay the same rates as other passengers:

‘Wheelchair users should pay the same amount what other people are paying. So, if the fare is R15 to town, wheelchair users should pay their fare of R15 with no exception. Because if you buy ice-cream you will have to pay for ice cream whether you are in a wheelchair or not.’ (TD3, Male, 48, Employed)

**Subsidies or contracts:** It was clear from the opinions of taxi drivers that they will only transport wheelchair users for free if they are compensated in another manner such as with subsidies or through a contract. A practical suggestion was to provide wheelchair users with tokens, thus allowing them to choose which taxi they want to use:

‘Maybe provide each one of them with 6 complimentary tickets for the month and they can decide what they want to use it for.’ (TD1, Male, 44, Employed)

Instituting contracts was another suggestion:

‘A minibus taxi organization should get a contract for five years to transport wheelchair users […] The reason for suggesting a five-year contract is to keep the specific minibus taxi organization accountable for the delivery of accessible minibus taxi services for wheelchair users.’ (S1, Male, 66, Retired)

However, co-researchers were worried that violence might ensue should such contracts be offered:

‘We are going to have a problem with minibus taxis drivers when it comes to a contract because when it comes to money, “Why did you get and why did I not get?” I can already see the minibus taxi war when it comes to who should get the contract.’ (TD5, Male, 48, Employed)

‘The co-researchers suggested that a tender process might reduce the risk of violence. The one solution to decide on who should get the contract is that there should be a tender process and the one who gets it is his job.’ (TD4, Male, 45, Employed)

**Drivers decide if and what wheelchair users pay:** The option of taxi drivers and taxi owners determining the amount wheelchair users should pay was discussed:

‘I think that owner of the minibus taxi should decide if a wheelchair user should pay or not pay.’ (C4, Male, 28, Unemployed)

Several ideas were shared regarding payment for minibus taxi services, including the option of free access to minibus taxi services. However, no definite conclusion was reached. Two main considerations were balanced during the discussion; on the one hand co-researchers recognised that minibus taxi drivers should be compensated for their services, on the other hand they called for financial assistance to make minibus taxi’s affordable for wheelchair users, especially those receiving social grants.

#### Theme 3: Facilitating ideal behaviour patterns

Co-researchers recognised how the attitude of one person can spill over and influence attitudes of those around them. They discussed ways to foster a collective effort including drivers, wheelchair users and fellow commuters to treat one another with respect, compassion and understanding in order to enhance the travel experience for everybody:

‘A little bit of positivity from minibus taxi drivers and passengers towards us as wheelchair users can go a long way. If minibus taxi drivers can provide the example of a more positive attitude to us this can lead that more passengers also becoming more positive towards us as wheelchair users.’ (WCU2, Female, 49, Cerebral vascular accident)

‘As minibus taxi drivers, we should know that we are here to provide a service for everyone and that we need to have a passion for our job and a positive attitude. There should be no discrimination towards anyone we are transporting.’ (TD4, Male, 45, Employed)

‘It should go both ways when it comes to positive attitude because when wheelchair users are positive and not arrogant, minibus taxi drivers and passengers will also be more positive and less arrogant.’ (TD2, Male, 32, Employed)

Co-researchers recognised that attitudes are dependent on knowledge and understanding. They felt that people who do not talk to each other cannot understand one another because the feelings and experiences of others are not known:

‘Some passengers believe a myth that if they get too close to wheelchair users or touch them, they might also end up in a wheelchair. Education is key.’ (WCU6, Male, 32, Spinal cord injury)

‘After I have been involved in this inquiry, I have a clear understanding about the dilemma of wheelchair users, and it will be a pleasure to transport them in the future. I will also encourage other passengers to accept wheelchair users as part of my clientele.’ (TD6, Male, 38, Employed)

Theme 3 emphasises the importance of respect between wheelchair users, passengers and minibus taxi drivers. Respectful interaction can be facilitated by the minibus taxi drivers who can set the tone on how wheelchair users should be treated in their vehicle. According to the co-researchers, minibus taxi drivers can play a role in the education of passengers and set the norm that all passengers, including wheelchair users, must be treated with respect and dignity.

#### Theme 4: Customised minibus taxis

With regards to fleet with different specs, co-researchers felt that the ideal scenario would be that some minibus taxis are adjusted to meet diverse user needs. The modification of minibus taxis are for example that some minibus taxis carry removable ramps, while others have a hydraulic lift and wheelchair docking station. Co-researchers also made design suggestions particularly focused on strategies for boarding and how best to secure wheelchairs during transit:

‘Minibus taxis should be horses for courses. We as wheelchair users have different diagnoses, so our needs for minibus taxi services will also differ. That is why it is important that there should be a fleet of minibus taxis which can accommodate wheelchair users with different transport needs. An example will be that a wheelchair user who is unable to do any transfers make use of an advanced minibus taxi that is equipped with a hydraulic lift system.’ (WCU9, Male, 37, Spinal cord injury)

‘I think the best thing for the future is that the minibus taxi has an automatic ramp where the wheelchair user can drive himself onto the minibus taxi and secure himself or herself. The wheelchair user will be independent and need not wait for the minibus taxi driver or guard for assistance.’ (TD1, Male, 44, Employed)

With regards the storage of wheelchairs inside the minibus taxis it requires securely and safe storage. However, it was considered a problem if the wheelchair took up space that could have been used by a commuter, in which case wheelchair users would be expected to pay an additional fee for the transport of the wheelchair. One suggestion was storing space can be behind the seat of the driver:

‘The wheelchair should be placed between the front and first set of rear seats.’ (TD2, Male, 32, Employed)

Another suggestion was a clip or frame at the back of the taxi:

‘I was just wondering if the wheelchair can be stored outside of minibus taxi at the back. Similar to what people are using for their bicycles; those bike frames.’ (S3, Male, 43, Employed)

Yet another suggestion involved removing some seats:

‘What about at the back of the minibus taxi if one removes the back seats of the minibus taxi it creates more space that can be used as storage for wheelchairs.’ (TD5, Male, 48, Employed)

Theme 4 shows that co-researchers felt that a fleet of minibus taxis with different specifications would best address the diverse transfer needs of wheelchair users. However, the cost implications and strategies to determine which modifications should be made by whom were not discussed.

#### Theme 5: Minibus taxi service delivery considerations

To ensure smooth use of minibus taxi services for wheelchair users, it is important that practical strategies are considered by minibus taxi drivers and wheelchair users alike. These include safe transfers, personal hygiene, suitable clothing, inclusiveness and home pickups and drop-offs.

**Handling of wheelchair users during transfers:** Co-researchers felt that the wheelchair user should direct the transfer. The importance of asking the wheelchair user how much and what type of support he or she needs during transfer was emphasised:

‘Before your transfer, any wheelchair user into or out of a minibus taxi you need to ask them permission if they need assistance and the type of assistance needed.’ (TD1, Male, 44, Employed)

Co-researchers also stressed that doing a transfer requires training:

‘Training and equipping minibus taxi driver and their guards is key.’ (WCU4, Male, 57, Cerebral vascular accident)

The co-researchers further suggested that educational tools showing transfer strategies could be made available:

‘I would also suggest that a pamphlet with photos and instructions should be developed which demonstrates step by step how transfers should take place when transferring wheelchair users into and out of minibus taxis.’ (S2, Female, 62, Retired)

‘I even have a better suggestion why don’t we make a video clip of how the transfers should take place and distribute it among the minibus taxi drivers as a reference they can use if they are unable to attend the training.’ (S3, Male, 43, Employed)

Wheelchair users who are women should feel comfortable when being assisted by men. Thus, co-researchers advised that training should include explicit guidance pertaining transferring women:

‘Minibus taxi drivers should know how to handle female wheelchair users; for instance, where they can be touched so that female wheelchair users don’t feel uncomfortable.’ (WCU7, Female, 54, Amputation of the lower limb)

Conversely, the point was raised that female wheelchair users’ choice of clothing influence the ease with which transfers can be made while maintaining their modesty; as such, they were advised to select their clothing with care:

‘It is important that wheelchair users and especially female wheelchair users wear appropriate clothing like a tracksuit for instance when they will be making use of minibus taxi services.’ (WCU2, Female, 49, Cerebral vascular accident)

**Personal hygiene:** Depending on the diagnosis of wheelchair users, some might not have bladder and/or bowel control. Having a bladder or bowel accident is embarrassing for the user and might elicit adverse reactions from fellow commuters and taxi drivers. Co-researchers felt trips should be organised with consideration of bladder and bowel schedules. In addition, precautions such as diapers and waterproof seat covers, were also proposed:

‘If possible, we need to prevent wheelchair users from these embarrassments, maybe wheelchair users should make use of diapers, or the minibus taxi seats should be covered with waterproof seats.’ (TD5, Male, 48, Employed)

**Inclusion of all stakeholders in planning of taxi services of wheelchair users:** Group members stressed the importance of involving all relevant stakeholders, including wheelchair users and caregivers, in planning of strategies for accessible minibus taxi services for wheelchair users:

‘As a minibus taxi driver, I realised again today the importance of inclusiveness or to communicate in a group like in this inquiry. If you as wheelchair users did not share your needs or bad experiences, I would never know the fear and anxiety you go through when using our services. I am very happy that you shared your transport needs with us, and I understand better how I can help you with transport […] More minibus taxi drivers and other stakeholders should be involved in discussions like these. Meeting wheelchair users and seeing their need will change their minds.’ (TD1, Male, 44, Employed)

**Home pickups and drop-offs:** For wheelchair users, the ideal scenario would be for minibus taxi services to pick them up and drop them off at their homes:

‘I had the most wonderful positive experience when the driver of a minibus taxi picked me up at my home. He was able to transfer me safely from my wheelchair into the minibus taxi and asked me if I am okay after he placed me on the seat.’ (WCU5, Male, 55, Spinal cord injury)

Theme 5 emphasised the need for collaboration in planning and executing of services, with wheelchair users guiding these processes.

## Discussion

This section explores the complexities that surround many of the proposed solutions. It also comments on the feasibility of the solutions based on available research evidence and current realities. Of note was that the co-researchers were mostly silent about who should take responsibility for the implementation of the suggestions they made. Furthermore, and of greater importance, they did not provide guidance on where the financial resources inherent to the success of many of the suggested solutions could be sourced from.

Suman and Patel (2022) explored the use of web directories, search tools of online websites that are smaller than search engines, for databases such as those suggested under Theme 1 ‘The ideal communication platform’. Web directories are created and maintained by human editors who include selected information and resources that adhere to a certain quality standard. They allow users to browse relevant information that is organised and structured alphabetically or subject-wise (Suman & Patel 2022). Databases, in the form of web directories, curated to contain the necessary information on taxi drivers (routes, accessibility features, training of driver, etc.) and wheelchair users (transfer needs, transit support needed, etc.) can help wheelchair users to identify and contact a minibus taxi driver of their choice. They can also provide minibus taxi drivers with information on the location of the wheelchair user and any specific accommodations they might require throughout the travel chain.

In order to develop such a directory, information must be gathered through a census. Following the initial development of a directory that is fit for purpose, the information must be updated regularly to ensure that new users and providers are added, address changes are noted and information of those who have moved or passed away is removed (Suman & Patel 2022). Initial development of the directory can be done in a way where specialist programming skills are not required for maintaining the directory; as such, wheelchair users could take responsibility for this aspect. Advocacy is required to convince local government or a local Disabled People Organisation to take responsibility for development and maintenance of a fit-for-purpose directory.

Co-researchers saw great benefit in being able to communicate with taxi drivers directly to secure pickup times and points. They initially proposed an online group for this purpose but reached consensus that their preference would be private communication. If cell phone numbers of minibus taxi drivers are made available to wheelchair users, both parties could communicate privately without the potential annoyances of higher data costs, unnecessary information, inappropriate postings and loss of privacy. It should, however, be noticed that this preference was strongly informed by the cohesion between taxi drivers and wheelchair users that developed through the cooperative inquiry process. As such, the preference for personal communication cannot be assumed to be transferable to other settings.

Establishing a system of communication between taxi drivers and wheelchair users to secure pickup times and points will reduce the health and safety risk associated with long waiting times (Gudwana 2019; Pretorius & Steadman 2018; Van Biljon & Van Niekerk 2021; Vincent & Chiwandire 2017). A similar service – Uber ASSIST – is already in existence. This service has three tiers with the lowest being Uber ASSIST, and then two more advanced levels. However, there are no Uber services available in the study area and Uber is more expensive than minibus taxis.

Theme 2, ‘Fair economical fares’, underscored the finding from previous studies that for minibus taxi drivers *time is money* and that ferrying as many people as quickly as possible remain their main concern (Cawood & Visagie 2015; Grut et al. 2012; Lister & Dhunpath 2016; Mudzi, Stewart & Musenge 2013; Venter et al. 2002; Vergunst et al. 2015). This has led to wheelchair users being left on the kerb or being exploited by having to pay for two or even three seats as their wheelchair takes up space. Furthermore, some of them must be accompanied by a caregiver (Chakwizira 2010; Gudwana 2019; Vergunst et al. 2015). The key message going forward is that wheelchair users must not be exploited, while minibus taxi drivers should not have to offer charity.

The price for minibus taxi services is dependent on the price of fuel. In South Africa, fuel prices are calculated monthly. At the time of the co-operative inquiry, the average minibus taxi prices for a return trip for commuters living in the study setting were as follows:

- R50 (or 2.75 USD or 2.46 EUR) to the day hospital.
- R28 (or 1.55 USD or 1.38 EUR) to the central business district.
- R28 (or 1.55 USD or 1.38 EUR) to Paarl Mall.
- R80 (or 4.47 USD or 3.94 EUR) inclusive of the travel fee for home pickups and drop-offs irrespective of where you wanted to go.

Table 4 provides a summary of costs if a wheelchair user from the study setting were to visit each of the above-stated places once a month. It shows that, depending on whether they paid for their wheelchair or not, as well as home pickups and/or drop-offs or not, they will need to budget between 10% and 69% of their monthly state disability grant of R2080 or 116.09 USD or 102.49EUR to cover these three trips. It’s important to note that the wheelchair users in this study could not travel without a carer as shown by the demographic information. The disability grant does not provide sufficient money to meet the needs of persons with disabilities. It is also often used to support others in the households as well. Thus, priorities are juggled to best balance expenses (Trafford 2023).

**TABLE 4:** A summary of cost of trips.

Co-research	Three trips’ usual fee	% of disability grant	Three trips’ home pick-up and drop-off fee	% of disability grant
Wheelchair user	R106	5	480	23
Wheelchair users and carer	R212	10	R960	46
Wheelchair user, carer and wheelchair	R318	15	R1440	69

Source: Trafford, Z., 2023, ‘“People don’t understand what we go through!”: Caregiver views on South Africa’s care dependency grant’, *African Journal of Disability* 12, a1114. https://doi.org/10.4102/ajod.v12i0.1114

The reality is that in most cases wheelchair users cannot afford minibus taxi services (which is a cheap form of public transport) for meeting even the most basic transport needs (Venter 2011). Many suggestions were made by the co-researchers about how services could be made more affordable; however, they did not reach consensus.

Lister and Dunpath (2016) recommended that minibus taxis owners should, such as buses, be provided with contracts with a route number, fixed times and a fixed fare system. Such contracts provide the opportunity to stipulate service-level agreements that include a commitment to transport wheelchair users, accessibility specifications for vehicles, relevant subsidy mechanisms and the relevant training required for safely assisting wheelchair users. Further investigation is required regarding the criteria used to issue such a contract.

The caution that implementing a system that utilises contracts or subsidies may lead to violence is well founded. The Paarl area is known for violence over minibus taxi routes and on several occasions the government had to intervene to stop fighting and killings over route turfs (Larner 2017; Lee 2012). Implementing subsidies is also a suggestion supported by Lister and Dhunpath (2016). It is favourable because it allows individual wheelchair users a choice of driver and taxi.

The establishment of a system using subsidies or contracts requires a dedicated budget, fair allocation processes and administration. Experience in other areas of South Africa has shown that putting these factors into place is fraught with challenges (Govender 2016). One of the biggest challenges will be allocating responsibility for the costs. Co-researchers suggested that the Department of Transport, Department of Social Services, Department of Health and Local Government should be responsible for the budget. Persuading these authorities to cover the budget will be challenging. Poor administration and logistical issues can also lead to service breakdown as shown by the challenges experienced by an STS operating close to Paarl in the City of Cape Town (Morta-Andrews 2018).

Another suggestion with regard to cost implications was that taxis should have different features to accommodate different physical needs of wheelchair users. The suggestion is supported by recommendations from Gudwana (2006), and similar strategies have been implemented in other countries (Park & Chowdhury 2018). However, there is little possibility of recovering the expense of vehicle modification and a scheme that covers such cost will be required. Conversely, minor modifications can be considered as a first step. A relatively minor modification that was strongly supported by co-researchers was the placement of handles at appropriate places inside the minibus taxi. These handles could give wheelchair users a secure place to stabilise themselves and thus decrease feelings of dependency, insecurity and anxiety during transfers and in transit, while simultaneously improving safety and the transit experience for all commuters.

Two of the suggestions made by co-researchers regarding the transit of wheelchairs are worth noting namely, securing the wheelchair at the rear of the taxi (such as a bicycle) or placing it behind the front seat. Neither of these solutions would require seating space; thereby removing the rationale for charging an additional fee for the wheelchair. Any negotiations or decisions regarding funding models for affordable access to taxi services for persons with disability (including wheelchair users) should include stakeholders with relevant power to affect the decisions made. When dealing with the taxi industry, the taxi owners are the relevant stakeholders.

A less tangible, but no less severe, obstacle was the hurtful attitude of drivers and fellow commuters towards disability. Lister and Dhunpath (2016) as well as Gudwana (2020) shared the opinion of co-researchers that workshops or awareness raising sessions should be a regular occurrence to educate the public at large on disability. They identified schools as starting points for education focused on disability inclusion. Gudwana (2020) recommended that educational programmes be extended to include roadshows and informal awareness campaign programmes at the minibus taxi ranks. Conversely, Mashiri et al. (2005) found that pamphlets and press releases to raise disability awareness led to little change in behaviour.

This suggests that the ways in which awareness raising campaigns are carried out needs to be modified and revised methods must be assessed, towards making them more effective.

Media campaigns on television, radio and advertisements have been used in some societies to facilitate behaviour change among fellow commuters and service providers (Murphy et al. 2006). Technologies such as persuasive design and game development have also been used to facilitate behaviour change among drivers and fellow commuters who discriminate against persons with disabilities (Consolvo; McDonald & Landay 2009).

Assisted transfers into and out of the minibus taxi requires physical touch, and for people to be in close proximity to one another. Co-researchers highlighted issues to consider during such transfers. The suggestion made included that users should direct the transfer process and indicate how and where they can be touched.

In the United Kingdom, training on disability awareness is provided by the Disabled Persons Transport Advisory Committee (2000). The Community Transport Association UK (CTA) has organised a driver training programme for minibus drivers to assess their ability to drive a minibus, as well as training them how to use equipment to transport wheelchair users during boarding with the use of boarding devices and wheelchair restraint systems (CTA 2000). The training is based on the Social Model of Disability and covers aspects such as etiquette and language. The trainer in these sessions called Disability Equality Training is often a person with a disability. In the Netherlands, 3.5 hours of disability awareness training was provided to the staff of public transport companies by a person with disability with traveling experience. In Scandinavian countries such as Finland and Sweden, disability awareness training is compulsory for taxi drivers and a Code of Practice has been issued for taxi drivers in Northern Ireland (Galvin 2017). With regard to disability awareness training, the emphasis needs to be on changing negative attitudes, communication and raising awareness of environmental and organisation barriers experienced by persons with disabilities (Fisher & Purcal 2017).

The co-researchers and previous research suggested that it is important that minibus taxi drivers should be equipped with knowledge and skills on how to handle wheelchair users (Duri & Luke 2022). In fact, all members of the travel chain such as the public, transport operators, passengers and the conductors need to be trained (Lister & Dhunpath 2016). Some efforts were made on a voluntary basis to train South African taxi drivers, but it is unclear how and where this training happened (Venter et al. 2002).

The topic remains a complex one. No easy solutions come to mind. Complex societal challenges, with solutions that require structural changes in key areas of society, can benefit from a socio-technical transition approach (Geels 2002; Kemp Avelino & Bressers 2011). Socio-Technical Transition Theory (STTA) provides a framework to facilitate major, often resisted shifts in an existing socio-technical system – ‘a cluster of aligned elements including technology, regulations, consumer practices, cultural meanings, markets, infrastructure, scientific knowledge, supply and maintenance networks’ (Schwanen, Banister & Anable 2011:1003). The STTA has been used in diverse sectors such as in agriculture, mobility (transport), energy and water (Geels 2019; Whitmarsh 2012).

Because of lock-in mechanisms such as behavioural patterns, sunk investments, infrastructure and dominant regulations in fields such as transport, socio-technical transitions do not occur easily (Geels 2010). Such lock-in mechanisms are seen in the challenges the South African minibus taxi industry experience in rendering equitable services to wheelchair users. Further work must be done to achieve a socio-technical transition during which the accessibility of minibus taxis for wheelchair users is transformed

### Strength and limitations

Co-operative group members were passionate about the topic and shared ownership of the process. Data were collected just after the lockdown for the coronavirus pandemic was ended in South Africa. This caused some wheelchairs users in the community to decide not to participate to protect their health. Consequently, the age range of the co-researchers was decreased. However, a spectrum of experiences was explored because of varying diagnosis and physical abilities among those who did participate.

## Conclusion

If governments want to achieve the goal of accessible transport for all, including wheelchair users, they should move away from vehicle-centred transport towards people-orientated mobility planning. The STTA provides a framework that can facilitate this shift to improve the accessibility of minibus taxis for wheelchair users and through that assist their occupational participation. The guidance of wheelchair users and other persons with disabilities should be sought throughout the transport chain. Current suggestions for solutions need further refinement. Accountability and funding sources remained particularly underexplored.

## Recommendations

Based on the findings of the study, following recommendations are proposed:

- Development of a web directory with relevant information as discussed should be further explored.
- Funding and subsidy models should be studied to determine the most suitable option for this and similar settings.
- Wheelchair users and disabled people’s organisations should inform the adaptations made to minibuses to improve access, such as where to place handheld supports.
- A proportion of designated taxis should have portable ramps and/or hydraulic lifts as well as a wheelchair docking station.
- Wheelchairs should be stored behind the front seat or at the back of the taxi and users should not incur extra costs for the wheelchair.
- All members of the travel chain should be trained with knowledge and skills regarding providing services and support to wheelchair users. Occupational therapists and health workers working at disabled people’s organisations could be involved in providing information, training, guidance, on optimal transfer strategies. A training manual could be developed based on the outcomes of this co-operative inquiry. This manual could be piloted in actual training minibus taxi drivers at no cost. They would receive a certificate or competence if certain minimum requirements are met.
